# Associations between emotions and psychophysiological states and confirmation bias in question formulation in ongoing simulated investigative interviews of child sexual abuse

**DOI:** 10.3389/fpsyg.2023.1085567

**Published:** 2023-03-28

**Authors:** Aleksandr Segal, Aistė Bakaitytė, Goda Kaniušonytė, Laura Ustinavičiūtė-Klenauskė, Shumpei Haginoya, Yikang Zhang, Francesco Pompedda, Rita Žukauskienė, Pekka Santtila

**Affiliations:** ^1^Department of Psychology, Mykolas Romeris University, Vilnius, Lithuania; ^2^Faculty of Psychology, Meiji Gakuin University, Tokyo, Japan; ^3^Faculty of Psychology and Neuroscience, Maastricht University, Maastricht, Netherlands; ^4^Faculty of Arts and Sciences, NYU Shanghai, Shanghai, China; ^5^School of Natural and Social Sciences, University of Gloucestershire, Cheltenham, United Kingdom

**Keywords:** child sexual abuse, emotions, GSR, heart rate, simulated interviewing, confirmation bias, investigative interviews, avatars

## Abstract

**Introduction:**

In forensic settings interviewers are advised to ask as many open-ended questions as possible. However, even experts may have difficulty following this advice potentially negatively impacting an investigation. Here, we sought to investigate how emotions and psychophysiological parameters are associated with question formulation in real time in an ongoing (simulated) child sexual abuse (CSA) interview.

**Method:**

In a experimental study, psychology students (*N* = 60, *M*age = 22.75) conducted two interviews with child avatars, while their emotions (anger, sadness, disgust, surprise and relief), GSR and heart rate (HR) were registered.

**Results:**

First, we found that general emotionality related to CSA and perceived realness of the avatars was associated with stronger overall emotional reactions. Second, we found that closed (vs. open) questions were preceded by more facially observable anger, but not disgust, sadness, surprise or relief. Third, closed (vs. open) questions were preceded by higher GSR resistance and lower heart rate.

**Discussion:**

Results suggest for the first time that emotions and psychophysiological states can drive confirmation bias in question formulation in real time in CSA.

## 1. Introduction

In abuse investigation settings interviewing is one of the crucial elements, especially if no other evidence is available, as usually is the case in alleged child sexual abuse (CSA) cases. Best practice recommendations advise interviewers to ask open-ended questions, such as “Tell me everything what happened!” “Tell me more about what happened yesterday!” “What happened after that?” (Ahern et al., 2018). Such questions are likely to elicit reliable, spontaneous and richer descriptions regarding past events, while closed questions are usually not recommended, because they tend to have the opposite effect (Lamb and Fauchier, 2001; Saywitz and Camparo, 2009). However, researchers have repeatedly demonstrated, that this recommendation is not sufficiently followed in practice (e.g., Baugerud et al., 2022, 2023) and interviewers tend to ask close-ended, leading and repeated questions (e.g., Santtila et al., 2004). Research suggests, that deviation from recommended guidelines for experts is at least party affected by their own cognitive biases, such as confirmation bias (Dror, 2020; Vredeveldt et al., 2022). Delving further into the mechanism of effect of confirmation bias and the factors related to it, some research suggests that emotions are involved in affecting interviewers’ bias, thus shaping the interviewers’ questioning style (e.g., Oxburgh et al., 2006; Ask and Granhag, 2007; Leach et al., 2022), abuse assumptions and conclusions (Zhang et al., 2022) and overall assessment of the interviews (Segal et al., 2023). Thus, in the present study, we were interested in studying the associations between emotions and confirmation bias by investigating differences in interviewers’ emotions and psychophysiological states preceding closed and open questions in real time during ongoing simulated CSA interviews.

In the present paper the terms open and closed questions were used for coding question types, where open questions encourage a free narrative and closed question prompt the interviewee to confirm or deny the statement provided by the interviewer or choose between provided options. Generally, open-ended questions are considered to be recommended, while close-ended questions are not recommended (Haginoya et al., 2021; Zhang et al., 2022). However, in the CSA interviewing context not all open questions are recommended. For example, questions about time (How often did it happen?) or open-ended questions that move the discussion from the reality to the fantasy level (What would have happened if he asked you to undress?) would not be recommended (see Table 1). Thus, even though the terms are closely related they are not completely synonymous.

**TABLE 1:** Question-type coding.

Category	Definition	Example
**Recommended questions**		
Facilitators	Open-ended and non-suggestive questions that elicit free narrative from children.	What happened after that?
Invitations	Non-suggestive questions that promote further narrative about the content previously mentioned.	Tell me everything what happened.
Directive	Questions that focus the children’s attention on the content the child has already mentioned for further explanation.	What do you do at home?
**Not recommended questions**		
Option-posing	Closed questions that focus the children’s attention on content that the child had not yet mentioned without implying a specific type of answer.	Were you doing something with him?
Specific suggestive	Questions that indicate what kind of answer is expected by assuming details that children have not mentioned.	I think someone did something bad to you. Tell me who was it?
Unspecific suggestive	Questions that indicate what kind of answer is expected without assuming details that children have not mentioned.	I heard from your mom that a guy did something bad to you. Is that true?
Repetitions	Questions continuously asking what the interviewer has already asked.	What are you always doing with XX? (after: What are you always playing with xx?)
Too-long/Unclear	Questions that ask for more than one detail at once. Also questions that contain words that are too difficult for the children’s cognitive level or that are grammatically unclear.	Where you with your dad, and what were you doing with him?
Multiple choice	Questions that focus the children’s attention on specific answers, or force them to choose among options.	Did you get hit on your face, your hands, or your feet?
Time	Questions that rely on temporal cognitive processes that are underdeveloped in children under 6 years of age.	When did mommy disappear from the park?
Fantasy	Questions that encourage children’s fantasies and may produce inaccurate answers.	If you were XX, what would you do?
Feelings	Questions that rely on emotional cognitive processes underdeveloped in children under 6 years of age.	Is mommy kind to you?

### 1.1. Challenges faced by interviewers

One would expect that trained professionals would be able to follow best interviewing practice guidelines and conduct unbiased interviews. However, field research has repeatedly shown that across countries, practitioners ask large proportions of option-posing and suggestive questions during forensic interviews (e.g., Korkman et al., 2006; Baugerud et al., 2022, 2023). More generally, forensic experts can be biased in terms of their observations, decision-making and question formulation, even without realizing it, and as a result an entire investigation can be impacted (Huang, 2021). In fact, even trained interviewers sometimes fail to follow best practice guidelines without continuous feedback and involvement in multiple training modules (Powell et al., 2008; Lamb, 2016) and tend to ask close-ended, leading, and repeated questions, thus increasing the likelihood of gathering only partial or even misleading information (Santtila et al., 2004; Bruck et al., 2006; Thoresen et al., 2009; Leach et al., 2022). To address these issues, great effort has been invested to design more effective interview training programs (e.g., Powell et al., 2014; Pompedda et al., 2015; Haginoya et al., 2020; Hassan et al., 2022) where continuing practice and ongoing feedback have been used to sustain the training effects (for a recent review, see Powell et al., 2022). Beyond the established efficacy of these training programs, serious gaming (Wouters et al., 2013) involving simulated avatar interviews (Pompedda et al., 2015) can be used as a tool to study the fundamental psychological processes, such as emotional impact on decision making during interviews (Zhang et al., 2022; Segal et al., 2023). For example, in an analysis of 2,084 simulated CSA interviews Zhang et al. (2022) found that interviewers’ preliminary assumption of sexual abuse having taken place predicted more frequent use of not recommended questions, bias toward a conclusion of abuse after the interview, higher confidence in the conclusion as well as a decreased likelihood of reaching a correct conclusion given the same number of available relevant details.

### 1.2. Bias in decision-making

As it may be expected from trained professionals to follow best practice guidelines in forensic interviewing, it may also be expected that expert decision-making should be immune to any subjectivity and would be based purely on scientific standards, latest research findings and generally on an unbiased approach (requirements, which are also documented in code of ethics and the laws regulating the work of specialists and experts). One might assume, that areas such as DNA testing or judicial decision-making are exactly those areas where no doubts should be raised regarding the decision and interpretation of the results. However, it was repeatedly demonstrated, that even such areas can be affected by biases that are inherent to humans (Ask and Granhag, 2005; Dror and Hampikian, 2011; Jeanguenat et al., 2017). Even judges who must be impartial and must make decisions purely based on the evidence that is presented to them are being affected by biases that are being influenced by their attitudes, ideology, backgrounds and previous experiences (Berryessa et al., 2023).

### 1.3. Emotions in decision making

A common idea prevails that in order for the legal system to function and the law to perform an objective function in the society, so that the public would trust and comply with the law – emotions must be “put aside” (Blix and Wettergren, 2016). However, when decisions are being made by human beings, it is doubtful that it is possible to “put” emotions aside, and thus it may well be assumed, that emotions will be experienced by the one who is making the decision as well as by those who are dependent on the decision. Thus, the role of emotions in decision-making should not be underestimated. Moreover, findings from areas related to forensic decision-making contexts (Dror et al., 2005; Charlton et al., 2010; Dror, 2020) support the hypothesis that interpretation and selection of information can be affected by emotional states. For example, in a study by Semmler and Hurst (2016) angry mock-jurors were more prone to carefully processing of evidence, detected more inconsistencies and recalled significantly more trial details as well as endorsed more of guilty verdicts.

### 1.4. Role of emotions in bias in forensic non-interview settings

Cognitive contamination or bias is present in all human beings, including forensic scientists (Jeanguenat et al., 2017). In a recent paper about sources of biases and fallacies, Dror (2020) explains, that experts may become biased due to various reasons, for example, reference materials, contextual information, organizational factors and others. Some of those sources of bias are related to emotional reactions by the experts, such as the data that possibly contains emotionally charged information (e.g., audio recording, that can reveal a brutal assault; gang-rape mixture DNA evidence) that can evoke experts’ emotions and subsequently impact decision-making. Further, Birze et al. (2022) reported, that professionals who analyze video evidence of violent crimes experience enduring impact of those videos. Constant viewing narrows the emotional proximity to violence and pushes to develop embodied reexperiences of the originally viewed traumatic events, for days, weeks, months and even more, following exposure (Birze et al., 2022). Thus, it is clear, that forensic experts are not immune to emotional impact (Dror and Hampikian, 2011), and it is important to acknowledge the existence of biases while not having a bias blind spot (Dror, 2020).

### 1.5. Role of emotions in bias in forensic interview setting with adults

Some research has demonstrated that emotions may influence the interviewer’s attitudes during the interview (Oxburgh et al., 2006). An analysis of thirty-four investigative interviews with alleged sex offenders revealed a significant effect of prior acquaintance with the victim in that a greater number of negative emotional utterances (e.g., contempt, anger, and disgust) were used by interviewers who had not previously interviewed the victim (Oxburgh et al., 2006). Other research on emotions in investigative interviewing context, indicated that interviewers’ emotional reactions affected how information was obtained from the interviewee as well as how the gathered information was assessed (Ask and Granhag, 2007; Magnusson et al., 2021). Lerner and Keltner (2000) described dimensions of anger that distinguish it from other negative emotions. Anger is characterized by appraisals of someone other than the self being responsible for a negative event; that the negative event was under some individual‘s control; and by certainty/or confidence about what happened (Lerner and Keltner, 2000). Opposite to anger, sadness is characterized by a greater sense of uncertainty and is more associated with situational causes (Ellsworth and Smith, 1988). Ask and Granhag (2007) also reasoned that people who feel angry tend to perceive another/specific person to be the cause of an unwanted event while feelings of sadness do not specifically lead to blaming someone else for what has happened. In their experiment, Ask and Granhag (2007) found that sad investigators - compared to angry investigators - analyzed case materials more thoroughly while the conclusions drawn by angry participants were not affected by the (in)consistency between suspects’ statements and the hypothesis. Magnusson et al. (2021) assessed the relationship between evoked emotions and interview tactics in suspect interviews in a sample of Swedish and Norwegian police and found that interviewers who experienced more negative emotions of anger, frustration and disgust employed more confrontational interviewing tactics such as emphasizing the seriousness of the crime, with the goal of obtaining a confession. This suggests that within an ongoing interview, we can expect the anger and disgust to be more likely to precede closed (suggestive) (vs. open) questions whereas sadness would be more likely to precede open (vs. closed) questions.

### 1.6. Role of emotions in forensic child interviews

Forensic child interviewing might give rise to emotions experienced by the interviewer, for example if the nature of the alleged crime is perceived as immoral and self-relevant in any way (Hutcherson and Gross, 2011). CSA is an emotionally charged topic not only for the public (McCartan et al., 2015) but also for professionals who interview allegedly abused children (Korkman et al., 2008; Magnusson et al., 2021). In a recent study Segal et al. (2022) explored how participants emotionally respond to child avatars with sexual abuse (vs. no abuse) scenarios. The main goal of that study was to examine whether participants respond emotionally to virtual child avatars with alleged CSA scenarios, and if they do, to analyze the patterns and differences of subjectively perceived feelings and objectively observed facial emotional expressions in response to either abuse or no-abuse narratives. In that study participants were presented with a number of child avatars describing either abuse or no-abuse details and their emotional responses were measured using automated analysis of facial expressions as well as self-reports. Self-report data revealed that participants reacted with greater relief when CSA was disconfirmed by the details revealed by the avatar but with stronger negative emotions of anger, sadness and disgust when CSA was confirmed. Although, the results from objectively expressed emotions were less clear. Segal et al. (2022) also expected surprise to be more likely after details disconfirming CSA. However, the difference in surprise in the two conditions did not reach statistical difference, however it was in the expected direction. Also, it was found that higher general emotionality related to CSA and higher perceived realness of the avatars made the differences clearer. Also, Segal et al. (2022) concluded that child avatar interviews are emotionally engaging and open a path to further research on the effects of interviewer’s emotions on interviewing. A second study (Segal et al., 2023) found a similar pattern of emotions in response to participants watching an actual dynamic interview. Participants responded with more sadness and disgust to confirmed CSA and with more relief to disconfirmed CSA interview outcomes. As expected by the authors, CSA scenario interviews were perceived as less suggestive than no-CSA scenarios. However, surprisingly, objectively more suggestive interviews were perceived as less suggestive and more appropriate b. This suggests that confirmation bias in the context of CSA interviews can have a seriously detrimental impact on the assessment of such interviews.

Emotions in the studies described above were elicited as a reaction to hearing a child avatar describe abuse or non-abuse details. However, the results may be relevant for formulating hypotheses about how emotions preceding a question could impact whether the question is biased and aims at confirming a CSA hypothesis or whether the question is open. Within the context of CSA interviews, we expected having an assumption of abuse, that is, thinking about CSA, to give rise to the same emotional pattern as listening to CSA details. In a corresponding fashion, we expected that thinking about the possibility of CSA not having happened would give rise to the same emotional pattern as listening to non-CSA details. We therefore expected that more of the negative emotions of anger and disgust would precede closed, CSA confirming questions while more of the positive emotions of relief and surprise would precede more open questions. Also, during an interview, CSA related details might be revealed by the avatar which would give rise to this emotional pattern reinforcing confirmation bias further which would in its turn again increase the probability of a closed question being asked next. Though, the expected role of sadness in this context seems somewhat unclear. On one hand, there is evidence of sadness resulting in more diligence and a stronger belief that the accused is guilty when analyzing the available data of an alleged crime (Ask and Granhag, 2007). Also, sadness is often accompanied with increased empathy (Nen et al., 2011) which is related to increased desire to help and support a victim (Bottoms, 1993; Huey and Kalyal, 2017). It can be assumed that if sadness is more related to the victim, then the interviewer will be more motivated to find support for the abuse hypothesis.

**FIGURE 1:**
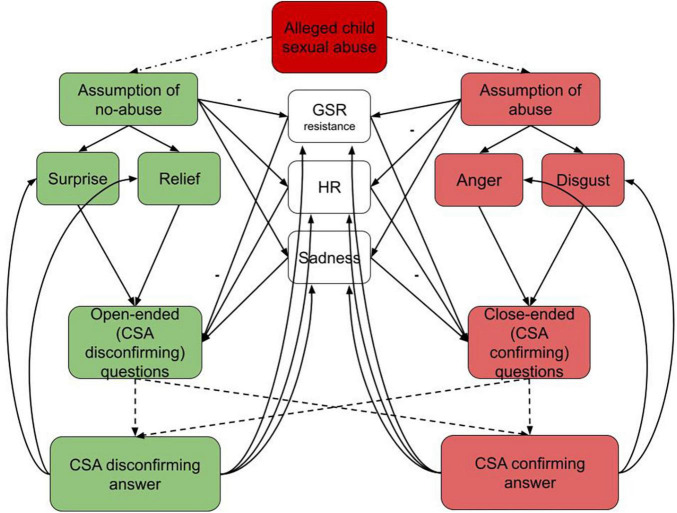
A preliminary model of the role of emotions on confirmation bias in question formulation in investigative child sexual abuse (CSA) interviews.

According to Arnold (1960) to arouse an emotion, an object or event must be appraised as affecting oneself personally as an individual with particular experiences and aims. Lazarus (1982) added that minimal information is needed to activate emotion which can be automatic and fast, and therefore not necessarily exceed the threshold of consciousness. Further, Lazarus (1966) defined the concepts of primary appraisal, secondary appraisal and reappraisal. Primary appraisal refers to the evaluation of a stimulus as significant to oneself. Secondary appraisal is related to coping potential: evaluation of the resources an individual has at their disposal to encounter with the situation. However, reappraisal may lead to changes of the primary and/or secondary appraisals. Based on appraisal theories of emotion (Arnold, 1960; Lazarus, 1966, 1982), our previous research (Segal et al., 2022; Zhang et al., 2022; Segal et al., 2023) and the literature mentioned above, we have drawn a preliminary model of the role of emotions and psychophysiological parameters on confirmation bias in question formulation in investigative CSA interviews (see Figure 1). A preliminary assumption of abuse is likely to result in more negative emotions of anger, disgust and sadness. Emotions of anger and disgust are associated with interviewers’ tendency to employ confrontational interviewing tactics and seeking for a confession of a committed crime. It suggests that these emotions would most likely reinforce the tendency to ask closed questions that support the abuse hypothesis, especially if the task demand components consume too much cognitive resources. Sadness on the other hand might result either in reinforcing the abuse hypothesis using closed questions when the feeling is directed toward the victim, or open-ended questions when the feeling is related to more openness regarding the overall case. On the other hand, an assumption of no-abuse would be associated with the more positive emotions of surprise and relief which are associated with alleviation of negative emotional state. Therefore, these emotions would be associated with less biased hypothesis testing.

Physiological parameters of heart rate (HR) and galvanic skin response (GSR) resistance, depending on their intensity might also influence either CSA confirming or CSA disconfirming questions (see Figure 1). It seems that lower HR (−) and higher GSR resistance lead toward CSA confirming questions, while higher HR and lower GSR resistance (−) lead toward CSA disconfirming questions. Further, we suppose that CSA confirming questions are more likely to elicit a CSA confirming response. However, there is a smaller possibility that a CSA disconfirming response might follow, thus changing emotional and psychophysiological reactivity of the interviewer.

### 1.7. Emotions, psychophysiology, and cognitive demands

As mentioned above, there are studies analyzing subjective and facially expressed emotional responses to CSA details revealed during interviewing. However, to our knowledge no studies have been conducted on interviewers’ psychophysiological responses and whether these could have an impact on subsequent question formulation in an ongoing CSA interview or in investigative interviews in general. However, given that emotions have psychophysiological correlates (Myroniv et al., 2017) and given that psychophysiological parameters are also related to stress and can especially be indicators of the cognitive demands of the task exceeding the available resources of a person (Jo and Kim, 2020), it is interesting to investigate whether they are related to an interviewer’s performance during an interview. In sum, previous research has not studied how psychophysiological parameters may affect question formulation during the interview process and what is their connection with confirmation bias in CSA interviewing.

It is known that physiological parameters, such as GSR (Shi et al., 2007) and HR reflect changes in the autonomic nervous system (Lee et al., 2006) and are valid indicators of stress, arousal levels and experienced levels of cognitive load (Healey and Pickard, 2005; Shi et al., 2007; Michels et al., 2013). Research indicates that GSR conductance (opposite to GSR resistance) can be a reliable measure of physiological stress (Han et al., 2020) and indicator of emotions, such as anger (Jha et al., 2018), sadness and happiness (Das et al., 2016). Feldman et al. (1999) meta-analytic study showed that one pathway through which stressors are thought to influence physiology is through their effects on emotion. Increases in negative emotions are associated with increases in cardiovascular (CV) responses to stress (Feldman et al., 1999). Research suggests that heart rate (HR) and skin resistance can be used as measures of worrying, that is, the higher the HR and the lower the skin resistance is (higher tendency to sweat), the higher levels of worrying it indicates and vice versa (Dua and King, 1987). Shirke et al. (2020) demonstrated that a sudden decrease in GSR resistance is associated with negative emotions while for positive emotions levels of GSR resistance tends to stay the same or change little in either direction. In any case, the changes are not sudden. Also, it is known that a lower level of anxiety (Chalmers et al., 2014) is associated with greater capacity for regulating emotional responding (Appelhans and Luecken, 2006). Some research has also shown that mean GSR conductance across studies increases as cognitive load increases (Shi et al., 2007). This leads to an assumption that when one experiences lower levels of anxiety and nervousness (associated with higher GSR resistance and lower HR) in combination with lower levels of cognitive capacity to elaborate on what actually happened in an alleged CSA situation, the interviewer might be more influenced by confirmation bias (higher levels of cognitive load), thus tempted to ask more closed questions confirming abuse hypothesis.

### 1.8. Simulated avatar interviews

Simulated avatar interviews were created as an alternative to theoretical training and practical training using adult actors to improve the quality of investigative interviews in alleged CSA cases (Santtila et al., 2012). This form of training, also known as serious gaming (Wouters et al., 2013) is a simulation training when training with real objects or human beings is expensive, risky or unethical (e.g., flying an aircraft, performing complex operations or interviewing allegedly abused children) and allows practitioners to acquire and improve complex practical interviewing skills in a cost-effective way and is potentially more scalable. When the training starts, the participants first read a background scenario concerning CSA allegations and afterward answer several questions regarding their preliminary assumption and level of certainty. Afterward, participants start to interview the avatar using natural spoken language. Each avatar has predefined either abuse or no-abuse scenario related memory contents that are (or are not) revealed according to the interviewers’ questions. Each question asked by the interviewer is categorized and coded by a trained human operator (Table 1) and afterward a probabilistic response algorithm associated with the question type determines the avatars’ response. If the interviewer asks a recommended (open) question the avatar is likely to provide a response containing a relevant or neutral detail. However, if the interviewer asks a non-recommended (usually closed) question, the avatar provides an affirmative or non-affirmative (yes or no) response with some of these responses being incorrect when compared to the memory contents of the avatar. Therefore, the interviewer can sometimes elicit a wrong detail in this situation. An operator also has the ability to manually provide additional narrative details unrelated to the suspected abuse labeled side details. After each interview participants provide a final decision about the presence or absence of abuse, their certainty level about the decision and a brief report of what they think has happened to the avatar (Pompedda et al., 2015; Pompedda, 2018; Zhang et al., 2022; Segal et al., 2023).

## 2. Current study

Simulated interviews with avatars about alleged CSA were used in the current study (Pompedda et al., 2015). These have the advantage of offering experimental control while still allowing the interviews to be interactive in a way that resembles actual interviews. However, the simulated nature raises the issue of the perceived realness of the interviews. In addition, there may be individual differences in how people react to the phenomenon of CSA that may be important to consider. Such general emotionality may influence the direction and intensity of emotional reactions (Cromer and Goldsmith, 2010) as well as decisions made in CSA cases (Bottoms, 1993) and higher perceived realism of the avatars may enhance these emotional reactions (Mohamad Ali and Hamdan, 2017; Segal et al., 2022).

The setup has 16 computer-generated child avatars with alleged sexual abuse scenarios. In the present study, we used two female child avatars with half of the participants in each group being presented with an abuse scenario avatar and then no-abuse while the other half were presented with the avatars in reverse order. During the simulation an operator coded the questions and depending on the question type the avatar provided a response as determined by algorithms reflecting real children’s behavior in interview situations (Pompedda et al., 2015). In this experiment, we used the Lithuanian version of simulated avatars.

### 2.1. Hypotheses

Based on previously discussed studies about interviewers’ emotional reactions, general emotionality, perceived realness, assessment of simulated CSA interviews with child avatars (e.g., Oxburgh et al., 2006; Segal et al., 2022), as well as interviewing tactics (e.g., Ask and Granhag, 2007; Magnusson et al., 2021) and psychophysiological responses to emotional stimuli (e.g., Shi et al., 2007) we formulated the following expectations:

(1) We expected general emotionality and perceived realness to predict overall stronger expressed emotions. We also investigated whether these variables would moderate the differences in the emotions and psychophysiological parameters preceding open and closed questions but we did not formulate any hypotheses concerning this given that these variables probably enhance the reactions themselves and therefore would not necessarily be expected to further moderate them.
(2) We expected closed (vs. open) questions to be preceded by more anger and disgust whereas we expected open (vs. closed) questions to be preceded by more surprise and relief. As the different theoretical models led to different predictions regarding the role of sadness, we did not formulate a directional hypothesis regarding it.
(3) We expected closed (vs. open) questions to be preceded by higher GSR resistance and lower heart rate.

Besides the *a priori* hypotheses, we explored gender differences and changes from first to the second interview. Here, our general expectation was that women would express emotions more strongly (Parkins, 2012) and that the strength of the expressed emotions would reduce from the first to the second interview because of habituation.

## 3. Materials and methods

### 3.1. Participants

The sample consisted of psychology students (*N* = 60 (47 women), age range 19–44 years, *M _*age*_* = 22.75, SD *_*age*_* = 6.74) recruited from Mykolas Romeris university. The invitation to participate in the study was sent out directly to all psychology bachelor students *via* e-mail. The data collection stopped when 60 participants took part in the study. None of the participants had any experience or training in child interviewing. Participants were granted a 10-euro shopping center gift voucher for their participation in the study.

The data of the present study were collected at the Applied Psychology Research Laboratory of Mykolas Romeris University in Lithuania by the first three authors. The study received ethical approval from the Ethics Committee for Psychological Research at Mykolas Romeris University, and documented in Decision Nr. 7/-2021.

### 3.2. Instruments

Participants completed the Cognitions and Emotions about Child Sexual Abuse (CE-CSA) (Gewehr et al., 2021) questionnaire which consists of three subscales: (1) Naive confidence (NC), (2) Emotional Reactivity (ER) and (3) Justice System Distrust (JSD). Responses were scored on a six-point scale ranging from 1 “*fully disagree*” to 6 “*fully agree*.” For the purposes of this study, we used only the emotional reactivity (ER) scale, which allowed us to measure baseline emotionality regarding CSA. The scale demonstrated good reliability (α = 0.88) and convergent validity [Emotional Reactivity subscale correlated positively and quite strongly with measures of empathy (*r* = 0.38, −0.42), and anger about sexual assault (*r* = 0.28)] (Gewehr et al., 2022). CE-CSA was originally created in German and translated into English by the authors of the questionnaire. CE-CSA in the present study was translated by the first author from English into Lithuanian and afterward it was back translated from Lithuanian to German to Lithuanian by another German-speaking colleague. When comparing the original German version and back translated German version the items were very similar.

Perception of avatar realism was measured using one-item, as in the previous experiments with Avatars on emotions (Segal et al., 2022). Participants were asked to answer how realistic they perceived the avatar to be. Responses were scored on a five-point scale ranging from 1 “*not realistic at all*” to 5 “*very realistic*.”

The whole study was designed and implemented using iMotions Biometric Research Platform 9.3 (iMotions, 2022) and every participant was recorded throughout the experiment. Afterward the video recordings were rendered and imported into Affectiva facial expression recognition engine, which is an integrated component of iMotions This software has built-in recognition for anger, sadness, disgust and surprise, but not for relief. For relief we extracted the most common facial action unit – AU12 Lip Corner Puller (Krumhuber and Scherer, 2011) which corresponds in iMotions to Lip Corner Depressor, and considered it as a facial reaction of relief. Emotion measurements do not depend upon a particular theory of emotion (Coppin and Sander, 2021).

Throughout the experiment the participants wore a portable Shimmer3 GSR + unit. The GSR sensor measures and captures GSR in real time. It monitors skin conductivity utilizing two reusable electrodes attached to two fingers of one hand. When stimuli activate the sweat glands, the increasing moisture will increase the conductance of the skin, allowing the current to flow more readily. Another reusable electrode was attached to the ear lobe and was used to capture Optical Pulse/PPG (Photoplethysmogram) that was converted to heart rate (HR). For the analyses of psychophysiological states, we used GSR and HR parameters.

For the analysis of facial and psychophysiological states we chose 5-s intervals prior to first five closed questions preceding a confirmatory answer and 5-s intervals prior to first five open questions preceding any relevant details provided as a response by the child avatar to the questions that were asked (Picture 1). For some of the participants the number of coded time intervals was lower due to them not asking enough questions. The same number of closed and open questions were included from the participant in these cases (see Table 2 for the numbers of coded questions).

**TABLE 2:** Number of coded questions in 1st and 2nd interview.

Number of coded open and closed questions per participant	1st interview	2nd interview
5 + 5	35	35
4 + 4	4	8
3 + 3	7	1
2 + 2	7	7
1 + 1	3	2
5 + 4*	1	
Total interviews**	57	53
Total questions	467	458
Total suggestive questions	233	229
Total non-suggestive questions	234	229
Average number of questions	8.19	8.48

*5 + 4 mistake in coding the number of open questions. **Number of interviews suitable for facial expression and psychophysiological data analysis out of 60 interviews.

**FIGURE 2:**
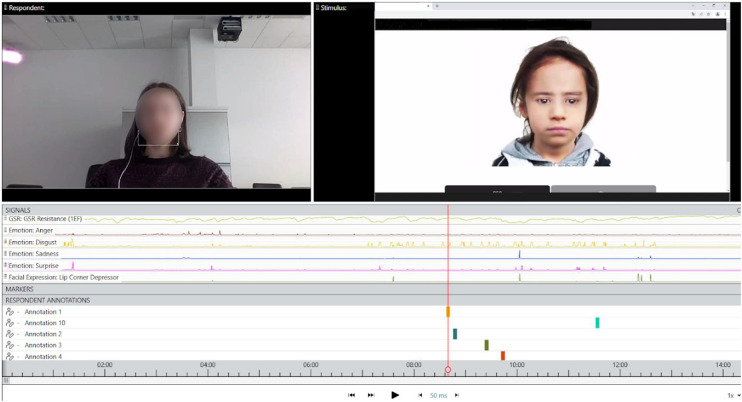
Coding of closed and open questions preceding a confirmatory answer.

Figure 2 shows that in real time a research participant conducting an interview with an avatar, while their facial expressions and psychophysiological indicators were captured on a storyboard. Later, iMotions allowed the overlaying of annotations of different time intervals on the timeline at points that will be used for further analysis. In our study, all annotations were of 5s length. Annotations in the timeline were placed prior to closed and open questions (max up to 5 + 5). The raw data showing the level of each facial expression and physiological indicators were later processed, extracting average means of each measured indicator per annotation.

By using measures mentioned above, we were able to gather facial expression recordings (objective) and psychophysiological measurements (objective).

### 3.3. Procedure

Upon arrival, each participant received a short explanation and instruction about the study. Participants were told:

*“The study is about interviewing allegedly sexually abused children using virtual child avatars and during the study you will have to fill in questionnaires about your emotional states, answer questions after watching an avatar speaking, then after watching a conducted interview with an avatar and finally you will have to interview two avatars that allegedly have experienced sexual abuse with the aim to figure out whether something has happened to them or not. During the whole experiment you will have to wear a portable sensor and you will be video recorded throughout the experiment. When you are presented with any stimuli, try not to cover your face with your hands.”*

Every participant signed an informed consent form before proceeding with the experiment. All data were collected in a laboratory setting and all of the questionnaires and measurements were conducted in the iMotions platform. After the completion of experiment, participants were debriefed with the interview outcomes and the aim of the study. Every participant then received a 10-euro gift coupon. The experiment included also other components not reported in the present paper.

### 3.4. Statistical analyses

Generalized estimating equations using the autoregressive relationship (AR) working correlation matrix to represent the within-subject dependencies were used to analyze the data. With this matrix, the repeated measurements have a first-order autoregressive relationship. It is defined so that the correlation between any two elements is equal to rho for adjacent elements, rho^2^ for elements that are separated by a third, and so on (IBM, 2021).

## 4. Results

### 4.1. Interviewers’ use of questions in the simulated interviews

For the first interview (Table 2), the overall mean was 7.70 (SD = 5.41) for the recommended and 17.79 (SD = 10.42) for not recommended questions (see Table 1); 1.47 (SD = 1.58) for the relevant, 1.77 (SD = 1.70) for the neutral and 3.98 (SD = 3.25) for the wrong elicited details; and 68.25% (SD = 15.60) confidence level regarding abuse assumption (*M* = 1.46; SD = 0.50). The overall number of correct decisions was 25 (Table 3).

**TABLE 3:** Correct and incorrect decisions in 1st and 2nd interview.

	1st interview		2nd interview		
	Correct decisions	Incorrect decisions	Correct decisions	Incorrect decisions	No answer
Total	25	35	34	23	3

For the second interview (Table 2), the overall mean was 6.54 (SD = 5.21) for the recommended and 18.72 (SD = 10.70) for the not recommended questions; 1.72 (SD = 2.21) for the relevant, 1.63 (SD = 1.82) for the neutral and 4.51 (SD = 3.48) for the wrong elicited details; and 64.04% (SD = 14.38) confidence level regarding abuse assumption (*M* = 1.51; SD = 0.50). The overall number of correct decisions was 34 (Table 3).

### 4.2. Gender and interview order effects

Before analyzing the potential impact of emotions and psychophysiological parameters on question formulation during the interviews, we explored possible gender and interview order effects on the measured variables. Generalized estimating equations controlling for dependence of measurements from the same participant and from the same interview were used when comparing male and female participants’ emotions and psychophysiological parameters. Women exhibited more sadness (*M* = 0.979, SE = 0.224) than men (*M* = 0.418, SE = 0.153), χ^2^ (1) = 4.261, *p* = 0.039 as well as more lip-corner depressor activity (relief) (*M* = 2.741, SE = 0.741) than men (*M* = 0.161, SE = 0.076), χ^2^ (1) = 11.994, *p* < 0.001. Women also had higher GSR resistance (*M* = 176.338, SE = 13.992) than men (*M* = 104.989, SE = 10.744), χ^2^ (1) = 16.358, *p* < 0.001. There were no significant differences in anger, surprise, disgust or HR.

Next, we used generalized estimating equations controlling for dependence of measurement from the same participants while comparing the parameters from the two interviews. The participants’ disgust levels reduced from the first interview (*M* = 1.107, SE = 0.436) to the second interview (*M* = 0.359, SD = 0.216), χ^2^ (1) = 4.200, *p* = 0.040. Their level of surprise also reduced from the first interview (*M* = 3.764, SE = 0.923) to the second interview (*M* = 1.097, SD = 0.318), χ^2^ (1) = 11.102, *p* < 0.001.

In contrast, the participants’ sadness increased from the first interview (*M* = 0.495, SE = 0.170) to the second interview (*M* = 1.273, SD = 0.338), χ^2^ (1) = 11.083, *p* < 0.001. Also, lip-corner suppressor activity (relief) increased from the first interview (*M* = 0.901, SE = 0.397) to the second interview (*M* = 3.711, SD = 1.161), χ^2^ (1) = 7.255, *p* = 0.007.

The participants’ GSR resistance reduced from the first interview (*M* = 169.119, SE = 17.313) to the second interview (*M* = 152.594, SE = 13.979), χ^2^ (1) = 11.082, *p* < 0.001. Also, their heart rate reduced from the first interview (*M* = 85.298, SE = 2.794) to the second interview (*M* = 79.244, SE = 2.876), χ^2^ (1) = 13.601, *p* < 0.001.

### 4.3. Correlations between general emotionality, perceived realness and facially expressed emotions and psychophysiological parameters between time periods preceding closed vs. open questions

Table 4 shows correlations between general emotionality and perceived realness and the emotion and the psychophysiological parameters as well as the inter-correlations between the latter variables. As expected, general emotionality was associated with more strongly facially expressed emotions. The associations were significant for all other emotions with the exception of sadness. General emotionality also had a negative association with heart rate. We also expected that perceived realness would be associated with more strongly facially expressed emotions. However, this was only true for surprise and lip-corner suppressor activity (relief). However, perceived realness was associated with both lower GSR resistance and lower heart rate. In terms of the interrelationships between the different emotions and psychophysiological parameters, we found that anger was positively correlated with both disgust and sadness. Further, sadness was strongly associated with lip-corner suppressor activity (relief), raising doubts about whether this measure is a valid indicator of relief. Finally, there were few associations between the psychophysiological parameters and the facially expressed emotions. Higher GSR resistance was associated with both more disgust and more lip-corner suppressor activity (relief).

**TABLE 4:** Correlations among study variables (*N* = 60).

	1	2	3	4	5	6	7	8
1. Emotional reactivity	–							
2. Perceived realism	0.160**	–						
3. Objective anger	0.073*	−0.028	–					
4. Objective disgust	0.108**	0.042	0.417**	–				
5. Objective sadness	0.063	0.058	0.270**	0	–			
6. Objective surprise	0.068*	0.199**	0.058	0.010	−0.050	–		
7. Objective relief	0.125**	0.159**	−0.019	−0.031	0.561**	0.014	–	
8. GSR resistance	−0.062	−0.109**	−0.013	0.107**	−0.035	0.058	0.088**	–
9. Heart rate	−0.069*	−0.167**	−0.019	−0.048	−0.034	0.016	−0.056	−0.012

**p < 0.01 and *p < 0.05.

### 4.4. Differences in emotions expressed on the face and psychophysiological parameters between time periods preceding closed vs. open questions

For the main analyses of interest, we again used generalized estimating equations controlling for dependence of measurement from the same participants and from the same interview. For each dependent variable, we first ran a Question Type × Gender analysis to understand whether closed and open questions were preceded by different types of emotional expressions or psychophysiological responses. After this, we ran Question Type × General Emotionality and Question Type × Perceived Realness analyses to see if the participants’ general emotionality regarding CSA or the extent to which they found the avatars to be convincing and realistic would moderate the associations.

**FIGURE 3:**
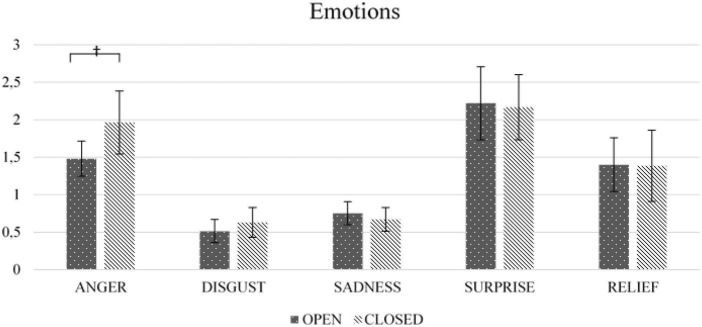
Differences in emotions preceding open and closed questions. ^†^ = 0.088. *N* = 60.

**FIGURE 4:**
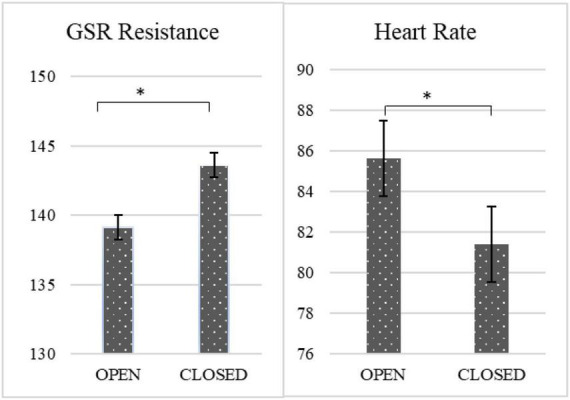
Differences in psychophysiological parameters preceding open and closed questions. *p < 0.05. *N* = 60.

Figures 3, 4 presents the differences in emotions and psychophysiological parameters preceding open and closed questions.

As expected, the participants expressed more anger in their faces preceding closed as opposed to open questions, χ^2^ = 2.917, *p* = 0.088. This effect was not moderated by gender (*p* = 0.216), general emotionality (*p* = 0.399) or perceived realness (*p* = 0.123). However, contrary to expectations, there were no differences in expressed disgust (*p* = 0.599), sadness (*p* = 0.416), surprise (*p* = 0.931), or lip-corner depressor activity (relief) (*p* = 0.959). Also, none of these effects were significantly moderated by gender (all *ps* > 0.316), general emotionality (all *ps* > 0.163) or perceived realness (all *ps* > 0.155).

As expected, the participants had higher GSR resistance preceding closed as opposed to open questions, χ^2^ = 11.350, *p* < 0.001. This effect was not moderated by gender (*p* = 0.132), general emotionality (*p* = 0.898) or perceived realness (*p* = 0.765). Also, as expected, the participants had lower heart rate preceding closed as opposed to open questions, χ2 = 13.496, *p* < 0.001. Also, this effect was not moderated by gender (*p* = 0.715), general emotionality (*p* = 0.345) or perceived realness (*p* = 0.261).

## 5. Discussion

The purpose of the current study was to investigate whether and how emotions and psychophysiological parameters are associated with question formulation in a simulated CSA investigative interview setting. Specifically, we aimed to measure objectively (by analyzing facial expressions) expressed emotions and registered psychophysiological parameters (GSR and HR) preceding open and closed questions and also to check whether the strength of these variables would be impacted by general emotionality and perceived realness. Results of the study provide, for the first time, evidence of the interplay between confirmation bias and emotions in CSA interviewers in real time. We found differences between open and closed questions for the emotion of anger, as well as for GSR and HR. Also, as expected, general emotionality and perceived realness of the simulation made some of the emotional reactions stronger.

This was the first time the Lithuanian version of the Avatar Training software was used. We compared the quality of the interviews with that of interviews in control groups of previous studies where no feedback or other intervention was provided (as in the current study no specific interviewing instruction nor feedback was provided). The current results correspond to the numbers reported by Pompedda et al. (2022) in a mega-analysis comprising of 997 control group interviews: proportion of recommended questions was about a third of all questions asked and use of recommended questions resulted in more correct and less incorrect information being elicited from the child avatars. This latter result means that the simulation worked as it should, consistent with what happens in interviews with real children (Phillips et al., 2012). In the current study, there was a minor deterioration of quality from the first to the second interview. In previous studies in control groups who receive no intervention, the performance typically stayed relatively stable over a number of interviews (Pompedda et al., 2022).

Looking into interviewers’ gender, we found that women had stronger emotional reactions of sadness and relief than men and also higher GSR resistance than men. For anger, surprise, disgust and heart rate there were no significant gender differences. These results are partially in line with Strongman (2003) findings of women being more emotional in expressing sadness. However, we did not find, as Strongman suggests, men to be more likely to express anger. Comparing emotional responses between interviews it was found that from the first to the second interview participants’ disgust and surprise levels reduced while sadness and relief levels increased. Physiological parameters of GSR resistance and heart rate also reduced from the first to the second interview. Observed changes in objective emotions might indicate that participants from the first to second interview show some level of either adaptation or desensitization. Reduced HR also supports the idea that after the first interview the participants felt less stress. However, the reduction in GSR resistance is not in line with this explanation.

Importantly and in line with Hypothesis 1, general emotionality regarding CSA as well as the extent to which the participants perceived the avatars to be realistic and believable were associated with stronger overall emotional reactions. This is in line with previous research with the avatars (Segal et al., 2022) suggesting that general emotionality and perception of realness are important factors contributing to reactions to (simulated) CSA interviews. Lehtonen et al. (2005) have suggested that in the field of virtual reality, aesthetics (which is defined as one’s emotional reactivity and feeling of presence and interaction with technology – a term that captures both emotional reactivity and feeling of realness) impacts learning and recall processes. They suggest that learners’ emotional reactivity adds to the cognitive load they experience (Chandler and Sweller, 1991) thereby potentially resulting in diminished learning capacity (Lehtonen et al., 2005) probably due to longer processing of information that elicits negative emotions. Our findings suggest that the avatars can be fruitfully used to study similar processes in CSA interviews.

In an earlier study of emotional reactions to listening to child avatars either revealing confirming CSA or disconfirming CSA details, Segal et al. (2022) found that self-reported emotions had a clearer pattern in line with the expectations (anger, sadness and disgust to confirmed CSA scenario details and relief to disconfirmed CSA scenario details) as opposed to facially expressed emotions (more anger to confirmed CSA vs. disconfirmed CSA details, while sadness, disgust, surprise and relieve were not significantly different in the facial expression analyses). The authors hypothesized that facially expressed emotional patterns should be clearer in a more dynamic interaction with the avatars, that is, that participants actually taking part in the interviews would display even clearer emotional patterns, however, this did not happen. We found that closed (vs. open) questions were preceded only by more facially observable anger, while there were no differences for disgust, sadness, surprise or relief. Thus, in this study we did not find the expected emotional pattern. In this way, Hypothesis 2 was only partially supported. We should consider at least three possible explanations: First, of course, that there are problems with the validity of analyzing facially recorded expressions (Kulke et al., 2020) and that a better solution than using iMotions facial recognition engine should be considered; second, that participants may feel that during serious interviews, such as CSA interviews, negative emotions should not be explicitly expressed (Oxburgh et al., 2006) so that they would not affect the interviewee’s responses; and we cannot reject the possibility that a person’s subjective experience of emotions is not well in line with their facially expressed emotions. The last explanation is in line with Izard (1997) who suggested that neuromuscular components of emotions do not always result in observable facial expressions. Thus, it is logical to think that the emotion that is most vividly observed might be the strongest of the ones that a person is experiencing at the moment.

In line with Hypothesis 3, closed questions were preceded by higher GSR resistance and lower heart rate than open questions. Higher GSR resistance basically means that the person is feeling less stressed (Villarejo et al., 2012). Also, lower HR means that a person is experiencing less stress (Dua and King, 1987). Some research indicates that lower levels of stress are associated with higher levels of self-confidence (Rees and Freeman, 2007). In an analysis of 2,084 simulated CSA interviews, Zhang et al. (2022) found that if the interviewer‘s preliminary assumption was that abuse had happened, the participant‘s confidence in this assumption was higher and more importantly, asked more closed questions later. This suggests that the participants who were less anxious tended to ask more closed-ended questions, which are more likely to lead to confirmation of a prior assumption, which in turn is related to poorer interview quality (more closed-ended questions, less useful and accurate information) and a greater possibility of reaching a false conclusion about the fact of the presence or absence of abuse.

Child sexual abuse interview itself is a complex task. It requires the interviewer not only to think about the question formulation, but also to evaluate child cognitive abilities to respond to the specific question. Another difficulty is related to truth estimation: the answers provided by the child need to be evaluated as true or false and whether they are in line with the question. And third, elicited details need to be evaluated in relation to the scenario the interviewer has in mind and evaluated for whether they support or disconfirm the abuse hypothesis. It should also be noted that all of these simultaneous processes are accompanied with emotional responses. According to Cognitive Load Theory (CLT) (Chandler and Sweller, 1991), the more a person is loaded with different task demand components that consume cognitive resources (i.e., attention, concentration, memory, thinking, awareness, etc.), the greater probability of mistakes, or in other words, lower quality of task performance. Some research also indicates that negative emotions have a direct correlation with a person’s attention span and may affect the cardiovascular system (Shirke et al., 2020). We hypothesize that up to a certain point the interviewer might be trying to handle the cognitive load and during this time physiological indicators can dynamically change (HR increase and GSR resistance decrease). However, after a certain point the interviewer may feel overloaded resulting in stronger negative emotional reactions and a reverse change of physiological parameters which would prompt one to choose the easiest path to continue with the task; in this case asking more close-ended questions. Such a line of questioning in light of reduced attention span would probably be reinforced by confirmation bias. In other words, higher GSR resistance and lower HR might be indicators of higher levels of cognitive load and related to confirmation bias.

It is also worth mentioning that correlations between the different emotion and psychophysiological variables were in line with the preliminary model of the role of emotions on confirmation bias in question formulation presented in the introduction. Positive correlations between anger and disgust and anger and sadness shows that these emotions go together as a group, while sadness also positively correlates with relief supporting the idea that the role of sadness in question formulation is ambiguous. Interestingly, general emotionality positively correlated with increased levels of all emotions except sadness. This supports the idea that more emotionally reactive interviewers should pay attention to their emotional reactions which can have an influence on question formulation in an ongoing interview. The last issue can be practically addressed, for example, by expanding the feedback in interview training to also focus on the role of emotions on confirmation bias.

The associations between the emotions and the psychophysiological parameters and the type of question asked were not moderated by either general emotionality or perceived realness of the avatars. This is not unexpected given that any impact these two factors seemed to be mediated by the emotions or psychophysiological parameters themselves and, in fact, this is what was found in the correlational analyses referred to above. The mediation account is supported by the previous study (Segal et al., 2022) that showed that these factors significantly moderated the emotional reactions to CSA details with individuals with higher emotionality having stronger emotional responses. In the current experiment, individual differences can thus be expected to have already had their impact through the variations in the emotions and psychophysiological parameters in influencing the type of question the interviewer asks next.

## 6. Strengths and weaknesses

The Avatar Training software is a promising way to study the questions addressed in the present research: the stimuli are standardized but still allowed a dynamic interaction process similar to that of an actual interview: the interviewers could formulate their questions freely and the child avatars also answered the questions orally. Also, we know from previous research (Segal et al., 2022) that the avatars invoke significant emotional reactions.

Secondly, to our knowledge, this is the first study to investigate the impact of emotions and psychophysiological parameters (GSR and HR) on question formulation in real time.

However, it is important to highlight that the sample was not a professional sample, thus their emotional reactions could be affected by lack of practical experience and knowledge about CSA interviewing. Also, it is important to notice, that this study could not prove any causal relationships between variables given that we did not use manipulations to cause changes in emotions and psychophysiology. However, we were interested in the natural interplay of emotional reactions rising during an interview and how they were associated with the following question type. That being said, there could be some unmeasured additional factor that explains the associations. This would most likely be some individual difference factor that explains both the emotional reactions and confirmation bias tendency.

## 7. Conclusions and future directions

In the literature, the problem of confirmation bias has been mostly looked at through the lens of cognition with notable exceptions. This also applies to the field of criminal investigations and investigative interviewing, again with exceptions (Powell et al., 2012; Zhang et al., 2022). Here, we demonstrated that emotions and related psychophysiological indicators are associated with question formulation in real time in simulated investigative interviews related to CSA. We also demonstrate that some of the emotional responses are driven by individual differences in general emotionality related to CSA. If the results of the present study are replicated (also in a professional sample), they have potentially important practical implications for the selection of personnel to work with CSA investigations as well as for training and supervision of such personnel.

People who are generally more emotional about CSA and have difficulties to restrain or regulate themselves emotionally might navigate the investigative interview in a more biased way in association with their expressed negative emotions. Further, CSA interview training could be enhanced with a short block of information regarding the link between emotions, physiology, questioning style and confirmation bias. Lastly, the supervision of CSA interviewing could be elaborated with feedback regarding emotionality and experienced emotions. That can be done at least in two ways: first - participants could be informed (or reminded) that negative emotions are associated with a tendency to formulate questions in a biased way, thus the interviewer should try to be aware of his/her own emotional state during the interview; second, if it will be possible, the interviewer could receive some feedback about his/her emotional state that can be observed by the supervising person. Also, interviewers, if needed, could additionally receive emotion regulation interventions in order to gain more self-control and become more self-aware about their emotional reactions during an ongoing interview. In addition, the approach we have used could potentially be used in other arenas as well. However, to be more certain about the clinical implications, we would want to replicate the findings in actual forensic child interviews.

## Data availability statement

The original data that support the findings of this study are available from the authors of the published articles upon reasonable request.

## Ethics statement

The studies involving human participants were reviewed and approved by the Ethics Committee for Psychological Research at Mykolas Romeris University. The patients/participants provided their written informed consent to participate in this study.

## Author contributions

AS: software, conceptualization, methodology, investigation, data curation, and writing – original draft preparation. AB and GK: investigation, data curation, and writing – review and editing. SH and FP: software and writing – review and editing. YZ: writing – review and editing. RŽ: project administration, funding acquisition, and writing – review and editing. PS: conceptualization, methodology, formal analysis, data curation, supervision, and writing – original draft preparation. All authors contributed to the article and approved the submitted version.
